# Phytochemical Profiling of Residual Leaves from an Alpine Landrace of Globe Artichoke (*Cynara scolymus* L.)

**DOI:** 10.3390/molecules30122649

**Published:** 2025-06-19

**Authors:** Marco Zuccolo, Angela Bassoli, Annamaria Giorgi, Luca Giupponi, Stefania Mazzini, Gigliola Borgonovo

**Affiliations:** 1 Centre of Applied Studies for the Sustainable Management and Protection of Mountain Areas—CRC Ge.S.Di.Mont., Università degli Studi di Milano, 25048 Edolo, Italy; marco.zuccolo@unimi.it (M.Z.); anna.giorgi@unimi.it (A.G.); luca.giupponi@unimi.it (L.G.); 2 Department of Agricultural and Environmental Sciences-Production, Landscape and Agroenergy—DiSAA, Università degli Studi di Milano, 20133 Milan, Italy; 3 Department of Food, Environmental and Nutritional Sciences (DeFENS), Università degli Studi di Milano, 20133 Milan, Italy; angela.bassoli@unimi.it (A.B.); stefania.mazzini@unimi.it (S.M.)

**Keywords:** artichoke leaves, “*Carciofo di Malegno*” landrace, cynaropicrin, caffeoylquinic acids, flavonoids

## Abstract

The globe artichoke (*Cynara cardunculus* L. var. *scolymus*) is a Mediterranean crop valued for its edible capitula and bioactive compounds. Post-harvest residual leaves are among the main by-products of artichoke cultivation and remain largely underutilized. This study reports a comprehensive characterization of the residual leaves of *Carciofo di Malegno*, an Alpine artichoke landrace. Comparative analysis was conducted against leaves from two commercial cultivars and a commercial herbal tea product. HPLC analysis revealed that *Carciofo di Malegno* exhibited the lowest levels of secondary metabolites. Cynaropicrin content was 0.52 ± 0.03 mg/g, lower than in the commercial samples, while the phenolic compounds were below the quantification limit. Proximate analysis indicated a distinctive nutritional profile, with significantly higher ash (8.01 ± 0.04%) and crude fiber (35.75 ± 0.29%) contents compared to all reference samples. These findings highlight the potential of *Carciofo di Malegno* residual leaves as a sustainable source of nutrients for functional food and nutraceutical applications. Their low content of bitter sesquiterpene lactones may enhance palatability, supporting their valorisation within circular economy frameworks. Moreover, their use may contribute to the in situ conservation of this landrace, reinforcing the link between agrobiodiversity preservation and the sustainable exploitation of agricultural by-products.

## 1. Introduction

The globe artichoke (*Cynara cardunculus* L. var. *scolymus* (L.) Fiori) is a perennial herbaceous plant belonging to the Asteraceae family. It most likely originated in the Mediterranean Basin, where it was domesticated from its wild ancestors [1]. Today, its cultivation is widespread across the region, making it a key element of the Mediterranean agricultural economy [2]. Indeed, although the globe artichoke is currently cultivated worldwide, more than 80% of global production still occurs in Mediterranean countries, with Italy being the second-largest producer, accounting for approximately 23% of global production [3].

The globe artichoke is an important component of the Mediterranean diet, appreciated not only for its culinary versatility but also for its remarkable nutritional profile, being an excellent source of inulin, dietary fiber, and essential minerals. Beyond its dietary significance, this plant has been traditionally employed in folk medicine for its hepatoprotective, choleretic, diuretic, and lipid-lowering properties [4]. These therapeutic benefits are primarily attributed to its high content of phenolic compounds, which contribute to its well-documented health-promoting effects [5].

The main phenolic compounds in the globe artichoke belong to the caffeoylquinic acid (CQA) family, along with *O*-glycosylated flavonoids such as derivatives of apigenin and luteolin [6]. Chlorogenic acid (5-*O*-caffeoylquinic acid) is the most abundant CQA derivative present in artichoke extracts, while cynarin (1,3-*O*-dicaffeoylquinic acid), although less abundant, is the most well-known compound of this class [4]. Cynarin, identified in both capitula and leaf extracts, is particularly noted for its choleretic and cholesterol-lowering effects [7].

Beyond phenolic compounds, globe artichoke is also a source of sesquiterpene lactones, a class of lipophilic metabolites primarily concentrated in the leaves, with lower levels detected in the capitula and stalks [8]. Among these, cynaropicrin stands out as the most abundant and is considered a chemotaxonomic marker of the species [9]. This compound has several bioactive properties, including anti-hyperlipidaemic, cytotoxic, and skin-protective activities [8]. Cynaropicrin is also one of the major contributors to the characteristic bitterness of globe artichoke, through the selective activation of the human bitter taste receptor hTAS2R46 [10]. The bitter taste recognition threshold for cynaropicrin is remarkably low, at 0.04 mM [11]. Chlorogenic acid is also described as bitter in sensory studies, with a recognition threshold of 28.22 μM [12].

The main commercial use of globe artichoke is the consumption of the immature inflorescence called capitulum or heads. Specifically, the edible portions of the capitulum consist of the inner bracts, the receptacle, the fleshy base of the outer bracts, and the upper portion of the floral stem [13]. As a result, artichoke cultivation generates a significant amount of biomass residues, with approximately 80–85% of the total plant biomass comprising residual materials [14,15]. A major component of this biomass is the residual leaves, which, along with the plant stalks, are often abandoned in the field after the harvest of the capitula [16]. However, within a circular economy framework, these residues can be repurposed for various applications, reducing waste and promoting sustainability. For instance, they can be utilized as animal feed or manure, serve as a source of biomass for bioenergy production [17,18], or be exploited for paper pulp production [19]. Additionally, these by-products offer potential for food-related applications, including as a source of milk-clothing enzymes for dairy production [17], as well as provide gelling agents for food applications and prebiotics [20,21]. Furthermore, they contain a rich array of secondary metabolites, including phenolic compounds and sesquiterpene lactones, similar to those found in the edible parts of artichokes [22,23,24]. Given this composition, artichoke by-products have garnered increasing interest as valuable raw materials for various applications, ranging from pharmaceutical formulations to health-promoting products and functional foods [25,26,27,28,29]. In particular, a recent review highlights the biological potential of globe artichoke extracts, especially those derived from leaves, in modulating oxidative stress and liver function, reinforcing their relevance in nutraceuticals and herbal preparations [30].

The valorization of artichoke biomass waste, in line with circular economy principles, could represent also a valuable strategy for promoting the conservation of agricultural biodiversity. In particular, it might support the sustainable utilization of local landraces, enhancing their preservation, and valorization, as well as encouraging resource efficiency in agricultural production [14,31].

A landrace is a locally adapted, traditional variety of domesticated plant species that has evolved over time through natural and human selection, exhibiting genetic diversity and resilience to specific environmental conditions [32]. Italy holds the most extensive collection of globe artichoke landraces, which have adapted to diverse environmental conditions across the country [5]. Nevertheless, many of these traditional varieties are being progressively replaced by more uniform and high-yielding cultivars, leading to a reduction in genetic diversity. Preserving and enhancing the value of landraces is therefore crucial to counteract genetic erosion and support sustainable agricultural systems [33,34].

In line with this perspective, we recently characterized *Carciofo di Malegno*, an artichoke landrace cultivated in the municipality of Malegno, Camonica Valley (Brescia, Italy). Traditionally grown on a small scale in terraced fields, *Carciofo di Malegno* is valued for its capitula, which are comparatively smaller than those of commercial cultivars [35].

In our previous work, we focused on the capitula—the only part of *Carciofo di Malegno* currently consumed—investigating their nutritional and phytochemical profiles. Currently, no data are available on the residual leaves, which represent a substantial but underutilized portion of the plant biomass. In this context, the present study specifically aims to characterize the phytochemical and nutritional features of *Carciofo di Malegno* residual leaves, focusing on the quantification of caffeoylquinic acids, flavonoids, cynaropicrin, and proximate composition parameters. A comparative analysis was also carried out using leaves from two commercial cultivars and a commercial herbal tea product. These data are relevant to evaluate the potential of these residual leaves, particularly in view of their valorisation within sustainable value chains, including the formulation of functional food and nutraceutical applications.

To date, studies exploring the reuse of artichoke by-products within circular economy frameworks have rarely focused on landraces. However, the valorisation of residual leaves could contribute to the in situ conservation of the landraces, reinforcing the link between agrobiodiversity preservation and the sustainable exploitation of local plant resources.

## 2. Results

### 2.1. Phytochemical Characterization of Artichoke Leaves

The phytochemical analysis of the artichoke residual leaves focused on the phenolic compounds and the sesquiterpene lactones. The main compounds of these classes were extracted and isolated from the herbal tea cut leaves (sample D).

For the phenolic fraction, mono-CQA chlorogenic acid (**1**), a small amount of the di-CQA cynarin (**2**) and the flavonoids scolymoside (**3**) and cynaroside (**4**) were isolated from the methanolic extract of the herbal tea cut leaves (sample D). Additionally, a third CQA derivative (**5**) was also obtained from the same extract. The identification of compound **5** as a constituent of sample D is described in Section 2.1.1.

For the sesquiterpene lactones, cynaropicrin (**6**) was isolated and identified as the main constituent from the acetone extract of sample D.

**Figure 1:**
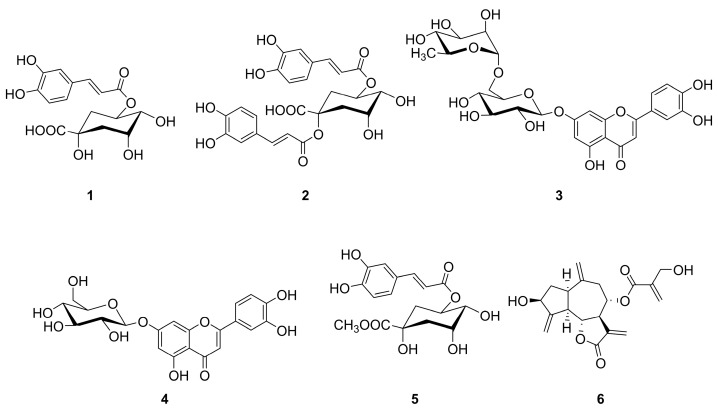
Structure of isolated compounds. Chlorogenic acid (**1**), cynarin (**2**), scolymoside (**3**), cynaroside (**4**), methyl chlorogenate (**5**), and cynaropicrin (**6**).

Compounds **1**–**6** were identified by NMR analysis through a comparison with literature data [36,37,38]. The structures of the isolated compounds are shown in Figure 1.

#### 2.1.1. Identification of Compound **5** as a Constituent of the Plant Material and Not an Extraction Artifact

The caffeoylquinic acid derivative **5** was identified as the methyl chlorogenate based on NMR analysis and comparison with literature data [37].

To exclude the possibility that compound **5** originated as an artifact during the acid treatment of the crude extract prior to chromatographic purification, a control experiment was performed. A pure standard of chlorogenic acid (**1**) was stirred in methanol at room temperature in the presence of Amberlite IR 120 (acidic form) under conditions analogous to those applied to the crude extract.

No formation of compound **5** was detected after 40 min to 4 h of treatment. Upon prolonged exposure (24 h), the degradation of chlorogenic acid was observed, and a thin-layer chromatography (TLC) spot of the caffeic acid methyl ester was detected, consistent with the occurrence of transesterification reaction. These findings indicate that compound **5** is unlikely to have been generated during the acid treatment of the crude extract.

To further assess the occurrence of compound **5** in the plant material, an ethanolic extract of sample D was prepared following the same extraction protocol used for compound isolation. Compound **5** was also detected in this ethanolic extract, confirming its presence in the original plant material and excluding its formation as an extraction artifact.

#### 2.1.2. HPLC Analysis of Artichoke Leaves Extracts

**Figure 2:**
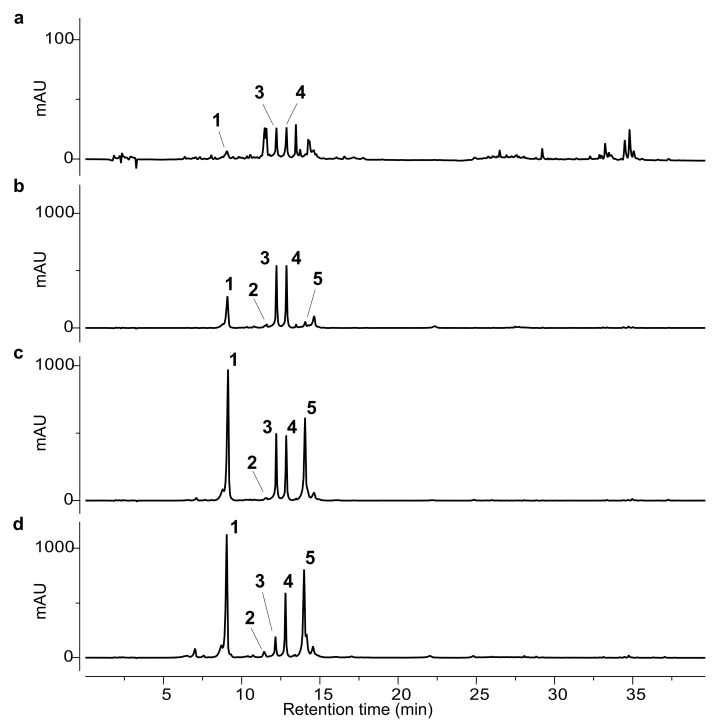
HPLC-UV chromatograms at 330 nm of ethanol extracts from samples A-D. (**a**) Sample A, upscaled 10-fold to enhance peak visibility; (**b**) sample B; (**c**) sample C; (**d**) sample D. The numbered peaks correspond to the following compounds: chlorogenic acid (**1**), cynarin (**2**), scolymoside (**3**), cynaroside (**4**), methyl chlorogenate (**5**).

**Figure 3:**
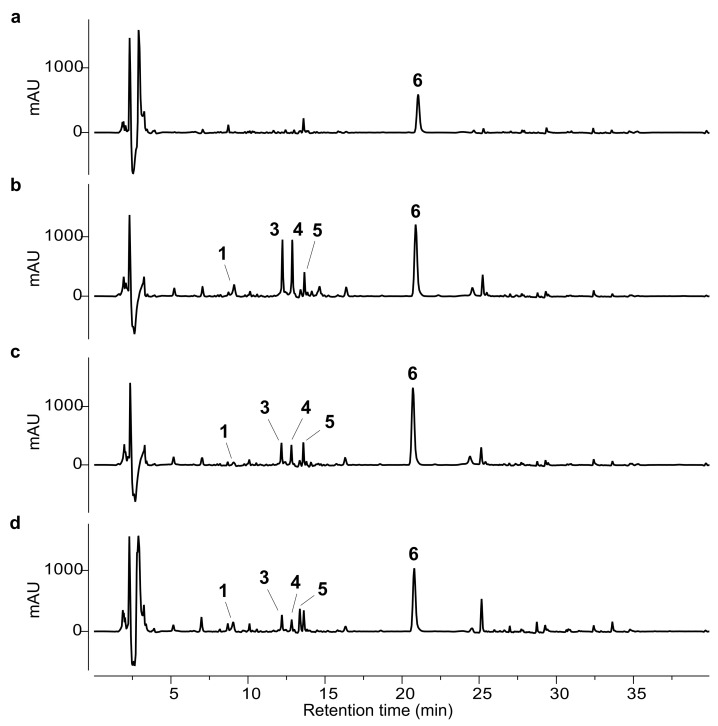
HPLC-UV chromatograms at 210 nm of ethanol extracts from samples A–D. (**a**) Sample A; (**b**) sample B; (**c**) sample C; (**d**) sample D. The numbered peaks correspond to the following compounds: chlorogenic acid (**1**), scolymoside (**3**), cynaroside (**4**), methyl chlorogenate (**5**), and cynaropicrin (**6**).

The isolated compounds (**1**–**6**) were used as external standards for the HPLC analysis. The phytochemical profiles of the ethanolic extracts obtained from the different artichoke leaf samples (A–D) are shown in Figure 2 and Figure 3.

The chromatographic analysis revealed marked qualitative and quantitative differences among the samples. Notably, the leaves of *Carciofo di Malegno* (A) exhibited a distinct chemical profile compared to the commercial cultivars (B–D). In sample A, chlorogenic acid (**1**) was detected only in trace amounts, with a barely detectable peak. The main constituents were the flavonoids scolymoside (**3**) and cynaroside (**4**), although their concentrations were lower than those observed in the other extracts. Several additional minor peaks were detected and tentatively attributed to flavonoid derivatives based on their UV absorbance spectra. However, their low abundance prevented isolation and full structural characterization.

In commercial samples B–D, chlorogenic acid (**1**) was generally the predominant compound, except in sample B, where flavonoids **3** and **4** were the main phenolic constituents. Scolymoside (**3**) and cynaroside (**4**) were present in comparable amounts in samples B and C, whereas in sample D, cynaroside (**4**) was more abundant than scolymoside (**3**).

Quantitative data are summarized in Table 1 and corroborate the qualitative trends observed in the chromatographic profiles. Among the analysed samples, *Carciofo di Malegno* (A) showed the lowest phenolic content, with all identified phenolic compounds below the limit of quantification. In contrast, sample D exhibited the highest concentrations for all quantified phenolics, except for scolymoside (**3**), which was significantly more abundant in samples B and C. Notably, sample B showed the highest accumulation of total flavonoid derivatives.

Cynarin (**2**) was present only in trace amounts across all samples, with the exception of sample A, where it was not detected.

Quantification of the methyl chlorogenate **5** revealed significant inter-sample variability. The highest concentration was observed in sample D, where compound **5** represented the second most abundant phenolic component. Sample C also contained appreciable levels of this compound, though substantially lower than those in sample D. Only trace amounts were detected in sample B, whereas it was not detected in sample A.

Regarding sesquiterpene lactones, no major qualitative differences were observed across the samples (Figure 3). Cynaropicrin (**6**) was the dominant representative of this class in all extracts. Sample A exhibited the lowest content of compound **6**, although not significantly different from that of sample B. Conversely, samples C and D contained markedly higher levels of cynaropicrin, with concentrations approximately twice those of sample B and about four times higher than in sample A.

### 2.2. Proximate Analysis

The proximate composition of the artichoke leaf samples (A–D) is summarized in Table 2. All samples exhibited comparable residual moisture, crude protein, and crude fat contents. In contrast, sample A showed a notably higher total ash content compared to the other samples, which displayed similar values. As for crude fiber, samples A and D had the highest levels, while samples B and C exhibited lower fiber contents.

## 3. Discussion

The present study provides a comprehensive characterization of artichoke leaf samples, integrating phytochemical and proximate analyses to evaluate both the secondary metabolite composition and overall nutritional profile. The investigation focused on the residual leaves of *Carciofo di Malegno* (sample A), a traditional landrace cultivated in the municipality of Malegno. To the best of our knowledge, this is the only documented case of an artichoke landrace adapted to a mountain environment. While the capitula are consumed, the leaves are typically discarded at the end of the production cycle, despite occasional uses as fodder, culinary herbs, or ingredients in herbal decoctions with reputed hepatoprotective effects (personal communication). The present findings contribute to the broader effort of valorising this local genetic resource through sustainable use of its agricultural by-products.

Comparative analysis was performed against waste leaves from two commercial artichoke cultivars (samples B and C), as well as a commercial herbal tea product derived from artichoke leaves (sample D). Phytochemical profiling specifically targeted phenolic compounds and sesquiterpene lactones, two classes of metabolites known to be characteristic of *Cynara cardunculus* [23,39].

Among the phenolic compounds, chlorogenic acid (**1**) represents the major caffeoylquinic acid derivative in *C. cardunculus*. This compound has been extensively studied for its multifaceted biological properties, including antioxidant, anti-inflammatory, antimicrobial, hepatoprotective, and metabolic-regulating effects [40,41]. In addition to its pharmacological relevance, compound **1** plays a central role in plant physiology. It accumulates in response to biotic stressors such as herbivory and fungal infection, contributing to the plant’s defensive mechanisms. Moreover, it acts as a scavenger of reactive oxygen species under abiotic stress conditions, thus participating in the mitigation of oxidative damage [42].

Another relevant caffeoylquinic acid derivative is cynarin (**2**), which, although not the most abundant in *C. cardunculus*, is one of the best-characterized. It is widely recognized for its choleretic and cholesterol-lowering effects and also exhibits hepatoprotective, antioxidant, anti-atherosclerotic, and immunomodulatory activities [7].

Other relevant polyphenolic constituents of *C. cardunculus* are scolymoside (**3**) and cynaroside (**4**), which are glycosidic derivatives of luteolin. Luteolin is a bioactive flavone that has been extensively investigated for its antioxidant and anti-inflammatory properties, which are mediated through the modulation of key cellular pathways involved in oxidative stress and immune responses [43,44]. In addition to these activities, luteolin has demonstrated anticancer potential by interfering with multiple processes such as apoptosis, cell proliferation, and angiogenesis [43,45]. Moreover, luteolin glycosides have shown antibacterial effects, including activity against phytopathogenic bacteria, suggesting a possible contribution to the plant’s defense system [46,47].

Among the sesquiterpene lactones, cynaropicrin (**6**) is considered a chemotaxonomic marker of the species and a major contributor to the bitter taste of the plant [9]. In addition to its well-documented anti-inflammatory and antiviral properties it has been investigated for potential anticancer effects through various mechanisms [48,49,50]. Cynaropicrin (**6**) also exerts potent antifeedant activity against several insect species, suggesting a role in plant defence against herbivory [9]. Moreover, cynaropicrin can act as an allelopathic compound by inhibiting seed germination and seedling growth in competing plant species [51].

The quantitative and qualitative analyses revealed notable differences in the metabolite profiles among the investigated samples. In particular, *Carciofo di Malegno* (A) displayed the lowest concentrations for all identified secondary metabolites. This reduced accumulation may be influenced by both varietal characteristics and environmental conditions. Environmental factors, such as altitude, temperature, and soil composition, are known to affect the biosynthesis and accumulation of plant secondary metabolites [52,53,54,55].

In addition to environmental and varietal factors, the developmental stage of artichoke leaves is also a well-documented determinant of phytochemical content. The material analysed in this study consisted of residual leaves harvested at the end of the production cycle, thus likely representing mature and physiologically senescent tissues. Leaf age and position within the plant strongly influence the phytochemical profile, with younger tissues generally exhibiting greater biosynthetic activity related to stress protection. Previous studies have demonstrated that younger leaves, particularly apical ones, are typically more enriched in phenolic compounds such as chlorogenic acid (**1**), cynarin (**2**), and flavonoid derivatives [56]. Similarly, cynaropicrin (**6**) content has also been reported to be higher in young leaves compared to older ones [57].

In this context, the low levels of secondary metabolites observed in *Carciofo di Malegno* leaves could reflect a combination of environmental adaptation, the physiological age of the material, and potentially intrinsic varietal traits. However, the current data do not allow us to disentangle the specific contributions of these factors. A comparative evaluation of *Carciofo di Malegno* cultivated under different agroecological conditions, including also ontogenetically younger leaves, may provide a more comprehensive assessment of the bioactive potential of this landrace.

Among the compounds isolated during phytochemical investigations, compound **5** was unambiguously identified as methyl chlorogenate. Methyl esters of CQAs are known to occur in several plant species, although they are frequently described as artifacts arising from extraction or purification processes [58]. Particular attention was paid to the use of Amberlite IR-120 resin, which has been previously employed to catalyse the esterification of caffeoylquinic acids [58].

To address this concern, we carried out a control experiment by exposing a methanol solution of chlorogenic acid to Amberlite IR-120 under conditions analogous to those used in the pre-treatment of the extract prior to chromatographic separation. No formation of the methyl ester was observed under these conditions, thus excluding the involvement of the resin in artefactual methylation.

Furthermore, compound **5** was consistently detected in crude ethanolic extracts. These findings suggest that the compound was already present in the plant material prior to the extraction process. To the best of our knowledge, this is the first report of methyl chlorogenate being detected in *C. cardunculus*.

Nonetheless, the possibility that it may originate from post-harvest transformations—such as drying or storage—cannot be ruled out. Further studies are required to verify the possible formation of this compound during post-harvest handling and to clarify its role within the overall phytochemical composition.

The proximate analysis highlighted some compositional differences between *Carciofo di Malegno* and the other artichoke samples. In particular, this landrace exhibited lower levels of crude protein and lipids, consistent with those observed in the other genotypes and aligned with previously reported values [59,60]. On the other hand, *Carciofo di Malegno* leaves showed higher levels of dietary fiber and ash. Among these parameters, the higher crude fiber content is particularly noteworthy. Dietary fiber is widely recognized for its important physiological functions, including the modulation of glucose absorption, cholesterol binding, and the promotion of intestinal health [61]. Artichoke-derived fiber, especially from agro-industrial by-products, has attracted increasing interest as a functional ingredient in food formulations. Fiber-rich flours obtained from artichoke waste have been successfully employed in the formulation of baked products, where they enhance dough hydration, extend shelf-life, and maintain acceptable sensory properties [62,63]. These applications underscore the potential of fiber-rich artichoke fractions to support sustainable food innovation [62,64].

Regarding ash content, it reflects a greater accumulation of inorganic substances and may indicate a higher content of mineral elements. However, further investigations involving elemental analysis are necessary to elucidate the actual mineral content.

While these compositional differences may be attributed to intrinsic varietal traits, environmental conditions and agronomic practices likely play a role and warrant further investigation.

Artichoke by-products, particularly post-harvest residual leaves, are increasingly recognized as valuable biomass for food, nutraceutical, and industrial applications. Their content of bioactive compounds, dietary fiber, and essential minerals aligns with circular economy principles and offers opportunities for sustainable reuse [22,25]. Such valorisation strategies may also contribute to the in situ conservation of traditional genotypes like *Carciofo di Malegno*, helping to safeguard agrobiodiversity and mitigate genetic erosion [14,31].

In this framework, the reuse of residual leaves from *Carciofo di Malegno* emerges as a promising opportunity. These leaves are typically discarded, with only limited and sporadic traditional uses. However, given their favourable nutritional profile—especially in terms of fiber and mineral content—*Carciofo di Malegno* leaves may be considered as potential ingredients for the development of innovative food formulations. The unexpected low concentration of cynaropicrin (**6**) and chlorogenic acid (**1**), two metabolites associated with pronounced bitterness [10,11,65,66], may be advantageous for improving palatability in food applications.

Taken together, the residual leaves of *Carciofo di Malegno* represent a potentially valuable, yet underutilized, resource. Although further research is necessary to fully evaluate their functional properties and application potential, their valorisation may offer a dual benefit: supporting sustainable use of agricultural by-products and contributing to the conservation of a traditional mountain-adapted artichoke landrace.

## 4. Materials and Methods

### 4.1. Plant Materials

Leaves of the *Carciofo di Malegno* landrace (sample A) were collected in June 2023 from multiple plants cultivated by the custodian farmer, Felice Pezzoni, in the municipality of Malegno (Camonica Valley; latitude 45°57′06′′ N, longitude 10°16′30′′ E, altitude 364 m a.s.l.). Sampling was performed at the end of the productive cycle, and leaves were excised at the base, near the main stem, from different locations within the cultivated plot to ensure representativeness.

The landrace has been morphologically characterized using the official CPVO TP/184/1 descriptors (Community Plant Variety Office), which are mandatory for the registration of new varieties. The corresponding documentation is provided as Supplementary Material. In November 2024, an application was submitted for the inclusion of *Carciofo di Malegno* in the Italian Register of Varieties of Agricultural and Food Interest (Law no. 194/2015), and the proposal received a positive evaluation by the Technical Committee of the Lombardy Region. The procedure is currently under review by the Italian Ministry of Agriculture, Food Sovereignty and Forests for official registration at the national level. To ensure long-term conservation and traceability, seed material was deposited at the germplasm bank “Centro Flora Autoctona-Parco Monte Barro” on 21 June 2023.

Leaves from two commercial globe artichoke cultivars (samples B and C) were obtained from a local street market, where they had been detached from the stems during pre-sale cleaning operations. Sample B was classified within the “Romaneschi” group, whereas Sample C belonged to the “Spinosi” type [67]. According to vendor information, both commercial samples were cultivated in Sicily. The botanical identity of both fresh samples was confirmed as *Cynara cardunculus* L. var. *scolymus* using the morphological identification keys reported in Flora d’Italia, 2nd ed. [68].

Additionally, a sample of dried artichoke leaves marketed as herbal tea (sample D, *Herbae*, Solimè) was purchased from a local herbalist shop.

Fresh samples (A–C) were dried in a laboratory oven (MPM Instruments M120-VF, Bernareggio, MB, Italy) equipped with forced-air ventilation, operating at 45 °C until constant weight was achieved. The residual moisture content of all samples, including dried fresh leaves (samples A–C) and sample D, is reported in Table 2, together with the other results of the proximate analysis described in Section 2.2.

The dried materials were manually fragmented and stored in screw-capped glass containers at room temperature and protected from light. The dried materials were finely pulverized using a vibrational mill (MM400, Retsch GmbH, Haan, Germany; frequency: 30 Hz; milling time: 1 min) and immediately used for the analysis. After grinding, the particle size of the samples ranged between 500 and 1000 µm.

### 4.2. General Experimental Procedures

All the solvents and reagents used were of analytical grade purity, while water was of HPLC-grade purity. All the solvents and chemicals were purchased from Merck (Milan, Italy) and used without further purification. Column chromatography was carried out on flash silica gel (Supelco (Milano, Italy), 230–400 mesh), while reverse-phase column chromatography was carried out on C_18_-silica gel for column chromatography (Merck, 230–400 mesh). Thin-layer chromatography (TLC) analysis was carried out on silica gel plates (Merck 60 F254) with visualization under a UV lamp (254 nm) and/or developed by heating after staining with cerium ammonium molybdate (12 g ammonium molybdate, 0.5 g ceric ammonium molybdate, 15 mL sulfuric acid, 235 mL distilled water).

### 4.3. Phytochemical Characterization of Artichoke Leaves

#### 4.3.1. Extraction and Isolation of Phenolic Derivatives (**1**–**5**)

The main caffeoylquinic acids and flavonoids were extracted by adapting the procedure described by Wang et al. [36]. Briefly, 100 g of powdered plant material was extracted with 1000 mL of 70% (*v*/*v*) aqueous methanol at room temperature under mechanical stirring for 3 h. The mixture was filtered under vacuum using a Büchner funnel, and the residue was re-extracted twice under identical conditions. The combined extracts were concentrated under reduced pressure to a final volume of 200 mL and subsequently depigmented by three successive liquid–liquid extractions with 200 mL of chloroform. The resulting aqueous layer was evaporated to dryness under reduced pressure (LABOROTA 4000eco, Heidolph Instruments GmbH & Co., Schwabach, Germany), affording a semisolid brown residue (32 g).

A portion of this residue (3 g) was suspended in 20 mL of methanol and treated with Amberlite^®^ IR 120 (acidic form, 200 mg) under stirring for 30 min to release acidic components from their salt forms. The resin was removed by filtration, and the filtrate was evaporated to dryness under reduced pressure. The crude extract was subjected to silica gel column chromatography, eluted with a gradient from 10:1 to 8:2 (*v*/*v*), consisting of ethyl acetate/toluene (9:1, *v*/*v*) and methanol/water (2:1, *v*/*v*), both containing 1% (*v*/*v*) formic acid. Fractions were combined based on their TLC profiles and further purified by Sephadex^®^ LH-20 column chromatography (20 g, 30 cm × 2 cm i.d.), eluting with methanol to afford pure compounds **1**–**5**.

#### 4.3.2. Extraction and Isolation of Cynaropicrin (**6**)

Cynaropicrin (**6**) was extracted by adapting the procedure described by Cravotto et al. [65]. Briefly, 30 g of the pulverized plant material was extracted three times with 300 mL of acetone at room temperature under mechanical stirring (IKA RW 16 basic, IKA, Staufen, Germany) for 3 h. The combined extracts were concentrated under reduced pressure to approximately 10 mL and then diluted with 50 mL of ethanol. The solution was treated with 50 mL of a 3% (*m*/*m*) lead (II) acetate solution to precipitate chlorophyll. After standing for 4 h, the mixture was filtered through a Büchner funnel, and the filtrate was concentrated under reduced pressure to half its original volume. The concentrated extract was diluted with 50 mL of water and then extracted twice with 100 mL portions of ethyl acetate. The organic phase was dried over anhydrous sodium sulfate, and the solvent was evaporated to dryness under reduced pressure, affording a semisolid brown residue (3.9 g).

The resulting crude extract was then fractionated by silica gel column chromatography using a hexane/ethyl acetate (7:3, *v*/*v*) mixture as the eluent to isolate cynaropicrin (**6**).

#### 4.3.3. NMR Analysis of Isolated Compound (**1**–**6**)

NMR spectra were recorded on a Bruker Avance 600 MHz spectrometer (Bruker GmbH, Mannheim, Germany), available at the Unitech Cospect platform at the University of Milan. The NMR spectrometer was equipped with a 5 mm triple-resonance probe with z-axis gradients and a variable temperature control unit. The analyses were performed at 25 °C. NMR samples were dissolved in deuterated methanol. Spectra were referenced to that of tetramethylsilane used as an external standard and taken as 0.0 ppm.

*3-O-Caffeoylquinic acid (chlorogenic acid)* (**1**): white amorphous solid (48 mg; purity 95%, HPLC); ^1^H NMR (MeOH-d_4_, 600 MHz) δ 7.58 (d, *J* = 15.8 Hz, 1H, H-7′), 7.06 (d, *J* = 1.5 Hz, 1H, H-2′), 6.97 (dd, *J* = 8.1, 1.5 Hz, 1H, H-6′), 6.78 (d, *J* = 8.1 Hz, 1H, H-5′), 6.28 (d, *J* = 15.8 Hz, 1H, H-7′), 5.35 (m, 1H, H-5), 4.17–3.90 (m, 1H, H-3), 3.73 (dd, *J* = 8.4, 2.9 Hz, 1H, H-4), 1.96–2.30 (m, 4H, H-2, H-6, overlapped signals). NMR data are consistent with the literature [36].
*1,5-Di-O-caffeoylquinic acid (cynarin)* (**2**): white amorphous solid (9 mg; purity 95%, HPLC); ^1^H NMR (MeOH-d_4_, 600 MHz) δ 7.48 (dd, *J* = 15.9 Hz, 1H, H-7), 7.47 (bd, *J* = 15.9 Hz, 1H, H-7), 6.92 (dd, *J* = 2.1, 0.5 Hz, 1H, H-2), 6.81 (dd, *J* = 2.1, 0.5 Hz, 1H, H-2), 6.75 (ddd, *J* = 8.2, 2.1, 0.5 Hz, 1H, H-6), 6.63 (d, *J* = 8.2 Hz, 1H, H-5), 6.59 (ddd, *J* = 8.2, 2.1, 0.5 Hz, 1H, H-6), 6.18 (d, *J* = 15.9 Hz, 1H, H-8), 6.51 (d, *J* = 8.2 Hz, 1H, H-5), 6.12 (d, *J* = 15.9 Hz, 1H, H-8), 5.37 (q, *J* = 3.5 Hz, 1H, H-3), 4.23 (ddd, *J* = 11.2, 9.5, 4.5 Hz, 1H, H-5), 3.62 (dd, *J* = 9.5, 3.7 Hz, 1H, H-4), 2.88 (dt, *J* = 16.0, 3.3 Hz, 2H, H-2a), 2.52 (ddd, *J* = 13.8, 4.5, 3.3 Hz, 1H, H-6a), 2.30 (dd, *J* = 16.1, 3.4 Hz, 2H, H-2b), 1.83 (dd, *J* = 13.8, 11.2 Hz, 2H, H-6b) NMR data are consistent with the literature [36].
*Luteolin 7-O-rutinoside (scolymoside)* (**3**): yellow amorphous solid (15 mg; purity 95%, HPLC); ^1^H NMR (MeOH-d_4_, 600 MHz) δ 7.39 (dd, *J* = 8.7, 2.2 Hz, 1H, H-6′), 7.37 (d, *J* = 2.2 Hz, 1H, H-2′), 6.82 (d, *J* = 8.7 Hz, 1H, H-5′), 6.75 (d, *J* = 2.1 Hz, 1H, H-8), 6.56 (s, 1H, H-3), 6.52 (d, *J* = 2.1 Hz, 1H, H-6), 5.05 (d, *J* = 7.3 Hz, 1H, H-1″), 4.74 (d, *J* = 1.5 Hz, 1H, H-1‴), 4.07 (d, *J* = 9.5 Hz, 1H, H-6″b), 3.93 (dd, *J* = 3.5, 1.6 Hz, 1H, H-2‴), 3.75 (dd, *J* = 9.5, 3.3 Hz, 1H, H-3‴), 3.29–3.70 (m, 7H, H-2″, 3″, 4″, 5″, 6″a, 4‴, 5‴), 1.18 (d, *J* = 6.2 Hz, 3H, H-6‴). NMR data are consistent with the literature [36].
*Luteolin-7-O-glucoside (cynaroside)* (**4**): yellow amorphous solid (22 mg; purity 95%, HPLC); ^1^H NMR (DMSO-d_6_, 600 MHz) δ 12.98 (s, 1H, OH-5), 7.46 (dd, *J* = 8.3, 2.0 Hz, 1H, H-6′), 7.43 (d, *J* = 2.0 Hz, 1H, H-2′), 6.92 (d, *J* = 8.3 Hz, 1H, H-5′), 6.79 (d, *J* = 1.9 Hz, 1H, H-8), 6.75 (s, 1H, H-3), 6.45 (d, *J* = 1.9 Hz, 1H, H-6), 5.09 (d, *J* = 7.3 Hz, 1H, H-1′′), 3.73–3.17 (m, 5H, H-2′′, 3′′, 4′′, 5′′, and 6′′). NMR data are consistent with the literature [36].
*3-O-Caffeoylquinic acid methyl ester (chlorogenic acid methyl ester)* (**5**): white amorphous solid (35 mg; purity 95%, HPLC); ^1^H NMR (MeOH-d_4_, 600 MHz) δ 7.53 (d, *J* = 15.9 Hz, 1H, H-7′), 7.05 (d, *J* = 1.5 Hz, 1H, H-2′), 6.96 (dd, *J* = 8.2, 1.5 Hz, 1H, H-6′), 6.80 (d, *J* = 8.2 Hz, 1H, H-5′), 6.24 (d, *J* = 15.9 Hz, 1H, H-7′), 5.30 (dd, *J* = 3.5 Hz, 1H, H-3), 4.16 (ddd, *J* = 3.5, 3.5, 1.5 Hz, 1H, H-5), 3.75 (dd, *J* = 3.5, 7.6 Hz, 1H, H-4), 3.72 (s, 3H, OMe), 1.96–2.28 (m, 4H, H-2, H-6). ^13^C NMR (MeOH-d_4_, 151 MHz) δ 175.7 (COOMe), 168.6 (CH=CHCO), 149.9 (C-4′), 147.5 (C=CCO), 147.1 (C-4′), 127.9 (C-1′), 123.3 (C-6′), 116.8 (C-5′), 115.4 (C-2′), 115.3 (C=CCO), 76.1 (C-1), 72.9 (C-3), 72.4 (C-4), 70.6 (C-5), 53.3 (COOMe), 38.3 (C-6), 38.0 (C-2). NMR data are consistent with the literature [37].
*Cynaropicrin* (**6**): white amorphous solid (382 mg; purity 95%, HPLC); ^1^H NMR (MeOH-d_4_, 600 MHz) δ 3.01 (dt, *J* = 10.8, 8.4 Hz, 1H, H-1), 2.12 (dt, *J* = 13.2, 7.2 Hz, 1H, H-2a), 1.74 (dt, *J* = 13.2, 7.2 Hz, 1H, H-2b), 4.53 (dd, *J* = 9.3, 7.1 Hz, 1H, H-3), 2.91 (dd, *J* = 10.4, 9.2 Hz, 1H, H-5), 4.37 (dd, *J* = 9.3, 7.1 Hz, 1H, H-6), 3.31 (t, *J* = 10.2 Hz, 1H, H-7), 5.15 (m, 2H, H-8), 2.42 (dd, *J* = 14.4, 3.6 Hz, 1H, H-9a), 2.75 (dd, *J* = 14.4, 5.2 Hz, 1H, H-9b), 5.66 (d, *J* = 3.2 Hz, 1H, H-13a), 6.14 (d, *J* = 4.0 Hz, 1H, H-13b), 4.93 (br s, 1H, H-14a), 5.17 (br s, 1H, H-14b), 5.35 (t, *J* = 1.6 Hz, 1H, H-15a), 5.45 (br t, *J* = 1.6 Hz, 1H, H-15b), 5.98 (d, *J* = 0.8 Hz, 1H, H-3′a), 6.32 (d, *J* = 0.8 Hz, 1H, H-3′b), 4.31 (s, 2H, H-4′). NMR data are consistent with the literature [38].

#### 4.3.4. Esterification Attempt of Chlorogenic Acid (**1**) to Methyl Ester (**5**)

Chlorogenic acid (**1**, 80 mg) was dissolved in methanol (20 mL), and 200 mg of Amberlite^®^ IR 120 (acidic form) was added to the solution. The reaction mixture was stirred at room temperature and monitored every 30 min by TLC. After 24 h, TLC analysis showed the complete disappearance of the starting material and the formation of a new product, which was identified as the methyl ester of caffeic acid.

#### 4.3.5. HPLC Analysis of Artichoke Leaves Extracts

The extracts for the High-Performance Liquid Chromatography (HPLC) assays were obtained by extraction with 75% (*v*/*v*) aqueous ethanol [39]. An exactly weighed sample (2 g) of pulverized artichoke leaves was transferred to a screw-top-cap conical-bottom centrifuge tube and extracted with 20 mL of solvent by stirring at room temperature for 30 min. The supernatant was collected by centrifugation (Hermle z300, HERMLE Labortechnik GmbH, Wehingen, Germany) at 4000 rpm for 10 min. The residue was then exhaustively extracted twice under the same conditions. The collected supernatants were pooled together and evaporated to dryness under reduced pressure at 45 °C. The residue was re-dissolved in methanol to a final volume of 5 mL.

For each sample, three independent extractions were prepared following the procedure described above. Each HPLC analysis was performed in duplicate.

The HPLC analysis for CQAs (**1**,**2** and **5**) and flavonoids (**3**,**4**) was performed using an Agilent 1200 series LC apparatus (Waldbronn, Germany) consisting of a degasser, a quaternary gradient pump, an auto-sampler, and an MWD detector. The detector channels were set at 210 ± 20, 250 ± 20, 290 ± 20, 330 ± 20, 370 ± 20, and 410 ± 20 nm.

A Luna^®^ 5 μm C_18_ (150 × 4.6 mm, 5 μm i.d.) column (Phenomenex, Torrance, CA, USA) at 40 °C was used for this analysis. Sample injections of 10 μL were made for all samples and standards. A binary gradient comprising 0.1% aqueous formic acid (*v*/*v*) (A) and acetonitrile (B) at a flow rate of 0.8 mL min^−1^ was used as the mobile phase. The gradient profile was as follows: 0 min, 5% B; 20 min, 25% B; 30 min, 95% B; and 40 min, 5% B. The absorbance wavelength was 330 ± 20 nm for caffeoylquinic acids and 370 ± 20 nm for flavonoids.

The HPLC analysis for cynaropicrin (**6**) quantification was performed using a PROSTAR liquid chromatograph (Varian, Palo Alto, CA, USA) comprising a ternary gradient pump and a UV detector. An ODS Hypersil C_18_ (250 × 4.6 mm, 5 μm i.d.) column (Thermo Fisher Scientific, Waltham, MA, USA) was used for this analysis. Sample injections of 20 μL were made for both samples and standards. A binary gradient comprising water (A) and methanol (B) at a flow rate of 0.5 mL min^−1^ was used as the mobile phase. The gradient profile was as follows: 0 min, 70% B; 10 min, 100% B. The absorbance wavelength was set at 210 nm.

Quantification was carried out using calibration curves prepared from standard solutions of isolated compounds **1**–**6**. Individual stock solutions were prepared in methanol at a concentration of 1 mg/mL. Working solutions were freshly prepared by serial dilution to obtain five calibration levels. For chlorogenic acid (**1**), scolymoside (**3**), cynaroside (**4**), and methyl chlorogenate (**5**), the concentrations used were 7.81, 15.62, 31.25, 62.5, 125, and 250 μg/mL. For cynarin (**2**), calibration levels were 5, 10, 20, 40, 80, and 160 μg/mL. For cynaropicrin (6), calibration was performed using the following levels: 0.15, 0.45, 0.75, 1.10, and 1.45 μg/mL. For each concentration level, three independent injections were performed, and the mean peak area was used to construct the calibration curves.

Calibration curves were obtained by linear regression analysis of peak area versus concentration. Quantitative results were expressed as mg per gram of dry weight (mg/g DW). The corresponding regression equations and correlation coefficients (R^2^) for each compound were as follows: chlorogenic acid (**1**) y = 28.545x + 198.980, R^2^ = 0.9971; cynarin (**2**) y = 33.992x − 4.421, R^2^ = 0.9984; scolymoside (**3**) y = 17.065x + 214.670, R^2^ = 0.9991; cynaroside (**4**) y = 28.668x + 958.21, R^2^ = 0.9983; methyl chlorogenate (**5**) y = 27.989x + 199.090, R^2^ = 0.9977; cynaropicrin (**6**) y = 37,299x − 1288.8, R^2^ = 0.9914. The analytical response was linear within the concentration ranges used for calibration for all analytes.

The limits of detection (LODs) and quantification (LOQs) were estimated based on a signal-to-noise ratio of 3 and 10, respectively, using the lowest standard concentration with detectable response. The signal-to-noise ratio was calculated from the peak height and the baseline noise measured in a blank region close to the retention time of each analyte. LOD and LOQ values were as follows: chlorogenic acid (**1**) and methyl chlorogenate (**5**), LOD = 2 μg/mL and LOQ = 6.7 μg/mL; cynarin (**2**), LOD = 3 μg/mL and LOQ = 10 μg/mL; scolymoside (**3**) and cynaroside (**4**), LOD = 0.5 μg/mL and LOQ = 1.7 μg/mL; cynaropicrin (**6**), LOD = 0.02 μg/mL and LOQ = 0.07 μg/mL.

### 4.4. Proximate Analysis

The proximate composition of dried artichoke leaves, including residual moisture, ash content, crude protein, crude fiber, and fat, was determined according to the methods described by AOAC International [69].

Moisture content was assessed by drying the samples in a ventilated oven (M120-VF, MPM Instruments, Italy) at 105 °C until constant weight (AOAC Method 930.15). Ash content was determined by incineration in a muffle (ZE muffle furnace, Ettore Pasquali s.rl., Milano, Italy) at 550 °C, according to AOAC Method 942.05. Crude protein was quantified using the Kjeldahl method (AOAC Method 945.18-B), using acid digestion, distillation, and titration of total nitrogen and applying a nitrogen-to-protein conversion factor of 6.25. Crude fat was extracted using a Soxhlet apparatus with petroleum ether as solvent, in accordance with AOAC Method 963.15. Crude fiber was measured following AOAC Method 962.09, by sequential digestion with diluted acid and alkali to isolate the indigestible fibrous fraction.

The total carbohydrate content was calculated as the difference of 100% minus the percentages of water, ash, protein, crude fiber, and fat.

All analyses were conducted on three independently prepared replicates.

### 4.5. Statistical Analysis

Data were analysed using one-way ANOVA (once the assumptions of normality of group data and homogeneity of variances were verified using the Shapiro–Wilk test and Levene’s test, respectively) with the Tukey test applied post hoc. The data are expressed as mean ± standard deviation (SD) and differences were considered statistically significant when *p* < 0.01. Statistical analysis was performed using the R software, version 4.4.2 [70].

## Figures and Tables

**Table 1:** Phytochemical analysis of artichoke residual leaf samples.

Component *		Sample			
		A (mg/g DW)	B (mg/g DW)	C (mg/g DW)	D (mg/g DW)
Chlorogenic acid	**1**	Trace	2.30 ± 0.08 c	6.90 ± 0.24 b	8.62 ± 0.82 a
Cynarin	**2**	n.d.	Trace	Trace	Trace
Scolymoside	**3**	Trace	5.31 ± 0.57 a	4.74 ± 0.42 a	2.15 ± 0.09 b
Cynaroside	**4**	Trace	4.29 ± 0.18 ab	3.99 ± 0.30 b	4.60 ± 0.05 a
Methyl chlorogenate	**5**	n.d.	Trace	4.87 ± 0.18 b	6.43 ± 0.74 a
Cynaropicrin	**6**	0.52 ± 0.03 b	1.11 ± 0.09 b	1.94 ± 0.67 a	2.36 ± 0.39 a

* Values are expressed as milligrams per gram of dry weight (mg/g DW). Data are presented as mean ± SD (*n* = 3). Different letters within the same row indicate statistically significant differences (*p* < 0.01, post hoc Tukey Test). Compounds reported as trace were detected at concentrations below the lower limit of the linear calibration range.

**Table 2:** Proximate composition and nutritional values of artichoke residual leaf samples.

Component *	Sample			
	A (%)	B (%)	C (%)	D (%)
Residual moisture	8.56 ± 0.05 a	8.49 ± 0.09 a	8.18 ± 0.11	8.45 ± 0.09 a
Crude protein	8.67 ± 0.08 a	8.61 ± 0.01 a	8.57 ± 0.04 a	8.59 ± 0.09 a
Crude fat	2.50 ± 0.03 a	2.48 ± 0.01 a	2.56 ± 0.02 a	2.45 ± 0.06 a
Ash	8.01 ± 0.04 a	6.75 ± 0.09 b	5.81 ± 0.05 c	5.61 ± 0.07 c
Crude fiber	35.75 ± 0.29 a	31.01 ± 0.11 b	31.93 ± 0.23 b	35.74 ± 0.39 a
Total carbohydrates	36.50 ± 0.29 b	46.67 ± 0.25 a	42.95 ± 0.21 a	39.16 ± 0.40 b

* Values are expressed as percentages (%), calculated per 100 g of dried material. Data are presented as mean ± SD (*n* = 3). Different letters within the same row indicate statistically significant differences (*p* < 0.01, post hoc Tukey Test).

## Data Availability

The data supporting the results of this study can be obtained from the corresponding authors upon reasonable request.

## Supplementary Materials

The following supporting information can be downloaded at: https://www.mdpi.com/article/10.3390/molecules30122649/s1.

## Abbreviations

The following abbreviations are used in this manuscript:

ANOVA	Analysis of Variance
AOAC	Association of Official Agricultural Chemists
CQAs	Caffeoylquinic Acids
HPLC	High-Performance Liquid Chromatography
LOD	Limit of Detection
LOQ	Limit of Quantification
NMR	Nuclear Magnetic Resonance
SEM	Standard Error of the Mean
TLC	Thin-Layer Chromatography
