# Supplementary material for: Phytochemical Profiling of Residual Leaves from an Alpine Landrace of Globe Artichoke (Cynara scolymus L.)
Source: Molecules. 2025 Jun 19;30(12):2649. doi: 10.3390/molecules30122649 (PMC12195720; doi:10.3390/molecules30122649)
Supplement: Supplementary file 1 [file molecules-30-02649-s001.zip › molecules-3662065-supplementary.pdf]

22 - CARCIOFO

(*Cynara scolymus* L.)

Scheda descrittiva morfologica

La descrizione dei caratteri indicati nella prima colonna con GIBA è raccomandata dal Gruppo di lavoro Biodiversità Agricola. La descrizione dei caratteri n. **9, 26, 27, 28, 41,** indicati nella seconda colonna con asterisco **(*)** è obbligatoria ai fini dell'iscrizione al Catalogo delle "varietà da conservazione".

N° GIBA	N° CPVO/ UPOV	CARATTERE	LIVELLO D'ESPRESSIONE DEL CARATTERE		VARIETA' DI RIFERIMENTO
1 GIBA	1	PIANTA: altezza incluso il capolino centrale (vedere figura seguente)	3	bassa	Violet de Provence, Tudela
			~~5~~ X	media 70-120 cm	Blanc Hyerois, Camus de Bretagne, Vertu
			7	alta	Caribou, Popvert, Salambo
2	2	PIANTA: numero di ramificazioni laterali sullo stelo principale	~~3~~ X	basso 1-3	Blanc Hyerois, Calico, Popvert
			5	medio	Salambo
			7	alto	Chrysanthème, Vertu
3 GIBA	3	STELO PRINCIPALE: altezza (escluso il capolino centrale)	3	basso	Capitan
			~~5~~ X	medio 60-110 cm	Castel, Salambo
			7	alto	Caribou

C= capolino centrale
L= primo capolino laterale

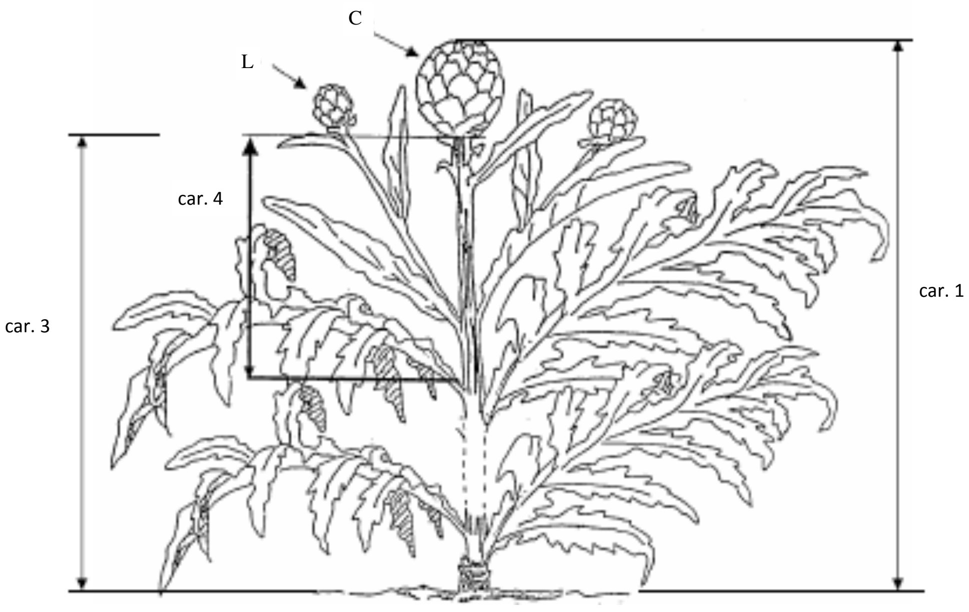

N° GIBA	N° CPVO/ UPOV	CARATTERE	LIVELLO D'ESPRESSIONE DEL CARATTERE		VARIETA' DI RIFERIMENTO
4	4	STELO PRINCIPALE: distanza tra il capolino centrale e la foglia più giovane ben sviluppata (vedere figura al car. 3)	3	breve	Caribou, Violet de Provence
			5 X	media 30-50 cm	Blanc Hyerois, Tudela
			7	lunga	Castel
5	5	STELO PRINCIPALE: diametro (a circa 10 cm sotto la base del capolino centrale)	3	piccolo	Violet de Provence
			5 X	medio 1,5-3,0 cm	Castel, Vertu
			7	grande	Carène
6 GIBA	6	FOGLIA: portamento (allo stadio di 10-12 foglie)	1 X	eretto	Capitan, Pètre, Vert de Provence
			3	semi-eretto	Calico, Camus de Bretagne
			5	orizzontale	Blanc Hyerois, Popvert
7 GIBA	7	FOGLIA: spine lunghe	1 X	assenti	Camus de Bretagne, Tudela
			9	presenti	Spinoso sardo
8	8	FOGLIA: lunghezza	3	corta	Tudela, Violet de Provence
			5 X	media 110-120 cm	Blanc Hyerois, Chrysanthème, Popvert
			7	lunga	Camus de Bretagne, Caribou
**9 GIBA**	**9 (*)**	**FOGLIA: incisioni (allo stadio di 10-12 foglie)**	**1**	**assenti**	**Tudela, Violet de Provence**
			**9 X**	**presenti**	**Camus de Bretagne, Vertu**
10	10	FOGLIA: numero di lobi 18 - 22	3	basso	Violet de Provence, Tudela
			5	medio	Blanc Hyerois, Chrysanthème
			7	alto	Salanquet
11	11	FOGLIA: lunghezza del lobo più lungo 20-35 cm	3	corto	Vertu
			5	medio	Orlando, Popvert, Sybaris
			7	lungo	
12	12	FOGLIA: larghezza del lobo più lungo 7-13 cm	3	stretto	Vertu
			5	medio	Orlando, Popvert, Sybaris
			7	largo	
13	13	LOBO: forma della punta (escluso il lobo terminale)	1 X	acuta	Camus de Bretagne, Vertu
			2	quasi ad angolo retto	Calico, Caribou, Salambo
			3	ottusa	

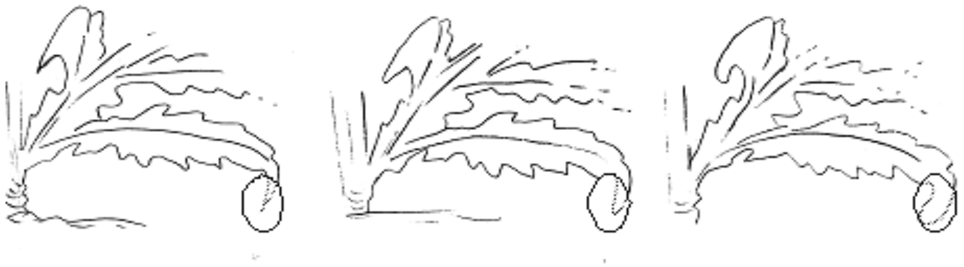

1 2 3

14	14	LOBO: numero di lobi secondari (sul 3°-4° giro di foglie) 6-11	1	assente o molto basso	Violet de Provence
			3	basso	Camus de Bretagne
			5	medio	Blanc Hyerois, Popvert
			7	alto	Orlando, Sybaris
			9	molto alto	

N° GIBA	N° CPVO/ UPOV	CARATTERE	LIVELLO D'ESPRESSIONE DEL CARATTERE		VARIETA' DI RIFERIMENTO
15	15	LOBO: forma della punta dei lobi secondari (come per 14)	1	molto appuntita	Vert de Provence
			~~2~~	appuntita	Blanc Hyerois, Tudela
			3	arrotondata	Cric, Popvert

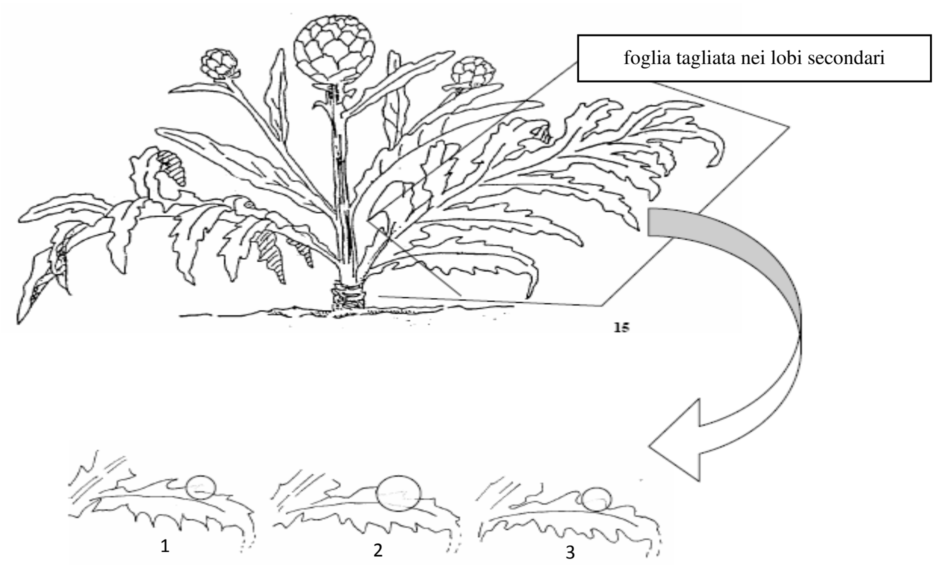

N° GIBA	N° CPVO/ UPOV	CARATTERE	LIVELLO D'ESPRESSIONE DEL CARATTERE		VARIETA' DI RIFERIMENTO
16	16	LEMBO FOGLIARE: forma in sezione trasversale	1	appiattita	Salambo, Vertu
			~~2~~	a "V"	Capitan, Castel
17	17	LEMBO FOGLIARE: intensità del colore verde (pagina superiore)	3	chiaro	Blanc Hyerois, Pètre
			~~5~~	medio	Violet de Provence, Tudela, Vertu
			7	scuro	Camus de Bretagne, Cric
18 GIBA	18	LEMBO FOGLIARE: sfumatura del colore verde	1	assente	Salambo
			2	giallastra	Blanc Hyerois
			~~3~~	grigiastra	Camus de Bretagne
19	19	LEMBO FOGLIARE: intensità della sfumatura grigia	3	debole	
			~~5~~	media	
			7	forte	
20 GIBA	20	LEMBO FOGLIARE: pubescenza della pagina superiore	1	assente o molto lieve	Camus de Bretagne, Castel, Vert Globe
			~~3~~	lieve	Vertu
			~~5~~	media	Carène, Popvert
			7	forte	Violet de Provence
			9	molto forte	
21 GIBA	21	LEMBO FOGLIARE: bollosità 3-8 bolle diametro: 0,5-1,5 cm	1	assente o molto debole	
			3	debole	Blanc Hyerois, Popvert
			~~5~~	media	Calico, Caribou
			7	forte	Chrysanthème
			9	molto forte	Cric
22	22	PEZIOLO: pigmentazione antocianica della base	~~1~~	assente o molto debole	Capitan, Carène
			3	debole	Castel
			5	media	Pètre
			7	forte	Violet de Provence
			9	molto forte	

N° GIBA	N° CPVO/ UPOV	CARATTERE		LIVELLO D'ESPRESSIONE DEL CARATTERE	VARIETA' DI RIFERIMENTO
23	23	CAPOLINO CENTRALE: lunghezza	3 X	corto 7-11 cm	Pètre
			5	medio	
			7	lungo	Vert de Provence
24	24	CAPOLINO CENTRALE: diametro	3 X	piccolo 4-7 cm	Vert de Provence
			5	medio	
			7	grande	Camus de Bretagne, Salambo
25 GIBA	25	CAPOLINO CENTRALE: taglia Peso: 30-100 g	3 X	piccolo	Vert de Provence, Violet de Provence
			5	medio	Blanc Hyerois, Chrysanthème
			7	grande	Castel, Salambo
**26 GIBA**	**26 (*)**	**CAPOLINO CENTRALE: forma in sezione longitudinale**	**1**	**tondeggiante**	**Castel, Green Globe**
			**2**	**ellittica larga**	**Chrysanthème, Vert de Provence**
			**3 X**	**ovale**	**Cric, Salambo**
			**4**	**triangolare**	**Tudela, Violet de Provence**
			**5**	**ellittica trasversale larga**	**Carène, Pètre**

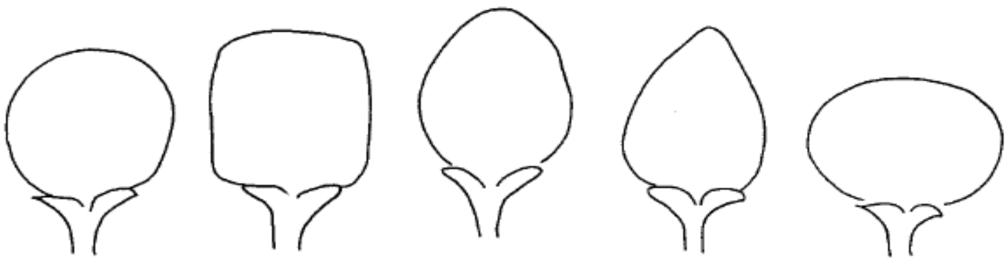

1 2 3 4 5

N° GIBA	N° CPVO/ UPOV	CARATTERE		LIVELLO D'ESPRESSIONE DEL CARATTERE	VARIETA' DI RIFERIMENTO
**27**	**27 (*)**	**CAPOLINO CENTRALE: forma della cima**	**1**	**appuntita**	**Violet de Provence**
			**2**	**arrotondata**	**Camus de Bretagne**
			**3 X**	**appiattita**	**Chrysanthème**
			**4**	**depressa**	**Carène, Pètre**
**28 GIBA**	**28 (*)**	**CAPOLINO CENTRALE: epoca di formazione** Da aprile a maggio/giugno (nell'area dove è tradizionalmente coltivato)	**3**	**precoce**	**Chrysanthème, Tudela**
			**5**	**media**	**Blanc Hyerois**
			**7 X**	**tardiva**	**Camus de Bretagne**
29	29	CAPOLINO CENTRALE: epoca di apertura Giugno (nell'area dove è tradizionalmente coltivato)	3	precoce	Chrysanthème, Vert de Provence
			5	media	Camus de Bretagne
			7 X	tardiva	Popvert, Tudela
30	30	PRIMO CAPOLINO LATERALE: lunghezza	3 X	corto 4-6 cm	Pètre, Popvert
			5	medio	
			7	lungo	Vert de Provence
31	31	PRIMO CAPOLINO LATERALE: diametro	3 X	piccolo 2,5-3,0 cm	Vert de Provence
			5	medio	Blanc Hyerois
			7	grande	Salambo
32	32	PRIMO CAPOLINO LATERALE: taglia	3 X	piccola	Violet de Provence
			5	media	Chrysanthème
			7	grande	Blanc Hyerois, Castel

N° GIBA	N° CPVO/ UPOV	CARATTERE		LIVELLO D'ESPRESSIONE DEL CARATTERE	VARIETA' DI RIFERIMENTO
33	33	PRIMO CAPOLINO LATERALE: forma in sezione longitudinale	1	tondeggiante	Castel, Salambo
			2	ellittica larga	Cric, Blanc Hyerois
			3	ovale	Velours
			4	triangolare	Violet de Provence
			5	ellittica trasversale larga	Pètre, Popvert

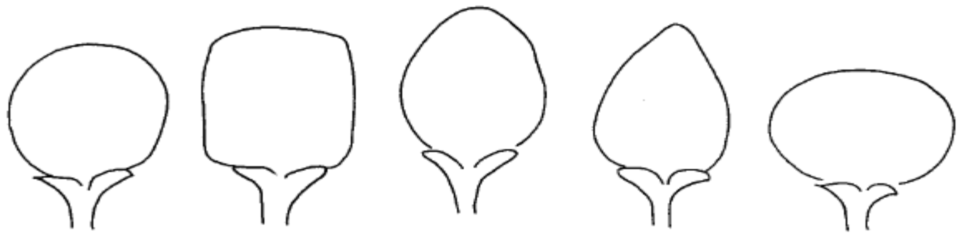

1 2 3 4 5

N° GIBA	N° CPVO/ UPOV	CARATTERE		LIVELLO D'ESPRESSIONE DEL CARATTERE	VARIETA' DI RIFERIMENTO
34	34	PRIMO CAPOLINO LATERALE: grado di apertura	3	debole	Salambo
			5	media	Blanc Hyerois
			7	forte	Chrysanthème
35	35	BRATTEE ESTERNE: lunghezza della base	3	corta	
			5	media	
			7	lunga	

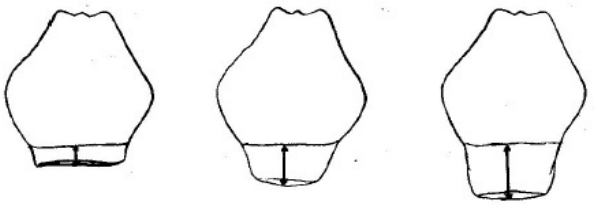

3 5 7

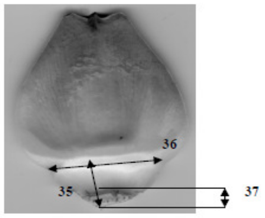

N° GIBA	N° CPVO/ UPOV	CARATTERE		LIVELLO D'ESPRESSIONE DEL CARATTERE	VARIETA' DI RIFERIMENTO
36	36	BRATTEE ESTERNE: larghezza della base	3	stretta	Orlando
			5	media	Blanc Hyerois, Popvert, Vertu
			7	larga	Pètre

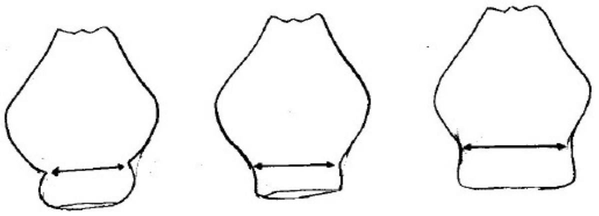

3 5 7

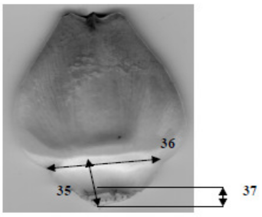

N° GIBA	N° CPVO/ UPOV	CARATTERE		LIVELLO D'ESPRESSIONE DEL CARATTERE	VARIETA' DI RIFERIMENTO
37	37	BRATTEE ESTERNE: spessore della base	3	fine	
			5	media	Blanc Hyerois, Popvert, Vertu
			7	spessa	Pètre

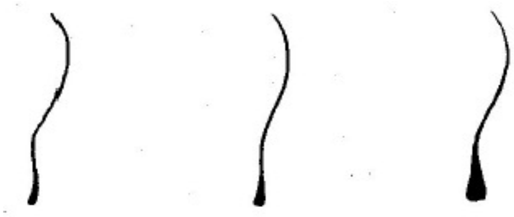

3 5 7

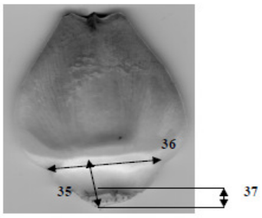

N° GIBA	N° CPVO/ UPOV	CARATTERE	LIVELLO D'ESPRESSIONE DEL CARATTERE		VARIETA' DI RIFERIMENTO
38 GIBA	38	BRATTEE ESTERNE: forma principale	1	più larga che lunga	Calico, Cric, Pètre
			2	tanto larga quanto lunga	Camus de Bretagne, Pètre
			3	più lunga che larga	Vert de Provence, Vertu
39 GIBA	39	BRATTEE ESTERNE: forma dell'apice	1	appuntito	Spinoso sardo
			2	appiattito	Talpiot
			3	depresso	Chrysanthème
40 GIBA	40	BRATTEE ESTERNE: profondità della depressione	3	superficiale 1-4 mm	Castel, Violet de Provence
			5	media	Blanc Hyerois
			7	profonda	Chrysanthème
**41 GIBA**	**41 (*)**	**BRATTEE ESTERNE: colore (faccia esterna)**	**1**	**verde** margini delle brattee talvolta violetti	**Blanc Hyerois, Tudela, Vert de Provence**
			**2**	**verde con striature violette**	**Violet de Provence**
			**3**	**violetto con striature verdi**	**Chrysanthème**
			**4**	**prevalentemente violetto**	**Cric, Salambo**
			**5**	**completamente violetto**	**Velours**
42 GIBA	42	BRATTEE ESTERNE: sfumatura del colore secondario (come 41)	1	assente	Calico
			2	bronzo	Blanc Hyerois, Sakiz
			3	grigio	Camus de Bretagne, Popvert
43	43	BRATTEE ESTERNE: curvatura della cima	1	assente	Castel, Salambo
			9	presente come fig. 9(a)	Chrysanthème (fig.a), Calico (fig.b)

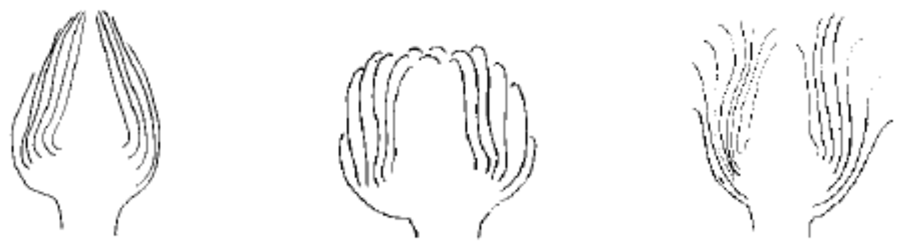

1 9 (a) 9 (b)

N° GIBA	N° CPVO/ UPOV	CARATTERE	LIVELLO D'ESPRESSIONE DEL CARATTERE		VARIETA' DI RIFERIMENTO
44 GIBA	44	BRATTEE ESTERNE: taglia delle spine	1	assenti o molto piccole	Calico
			3	piccole	Chrysanthème, Vertu
			5	medie	Violet de Provence
			7	grandi	
			9	molto grandi	Spinoso Sardo
45	45	BRATTEE ESTERNE: mucrone	1	assente	Chrysanthème, Pètre
			9	presente 0,5-1,5 mm talvolta spinoso	Camus de Bretagne

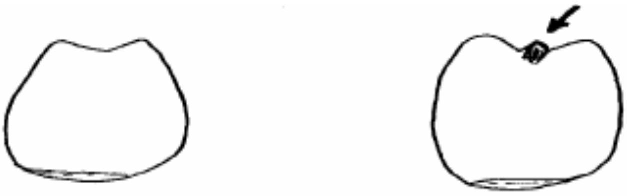

1 9

N° GIBA	N° CPVO/ UPOV	CARATTERE	LIVELLO D'ESPRESSIONE DEL CARATTERE		VARIETA' DI RIFERIMENTO
46	46	CAPOLINO CENTRALE: pigmentazione antocianica delle brattee interne	~~1~~	assenti o molto lieve (assente)	Popvert
			3	lieve	Castel
			5	media	Blanc Hyerois
			7	forte	Chrysanthème
			9	molto forte	Salambo
47	47	CAPOLINO CENTRALE: densità delle brattee interne	~~3~~	lasche	Camard, Calico
			5	medie	Camus de Bretagne
			7	dense	Cacique, Compact

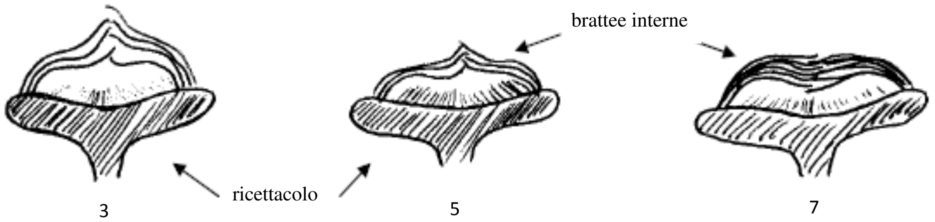

N° GIBA	N° CPVO/ UPOV	CARATTERE	LIVELLO D'ESPRESSIONE DEL CARATTERE		VARIETA' DI RIFERIMENTO
48	48	RICETTACOLO: diametro (vedere figura seguente) 2,5-3,0 cm	3	piccolo	Violet de Provence
			5	medio	Camus de Bretagne
			7	grande	Capitan, Salambo
49	49	RICETTACOLO: spessore (come per car. 48) 0,4-0,8 cm	3	fine	Blanc Hyerois, Tudela
			5	medio	Pètre
			7	spesso	Camus de Bretagne, Castel
50	50	RICETTACOLO: forma in sezione longitudinale	1	appiattita	Carène
			2	leggermente depressa	Camus de Bretagne, Salambo
			~~3~~	fortemente depressa	Blanc Hyerois, Chrysanthème

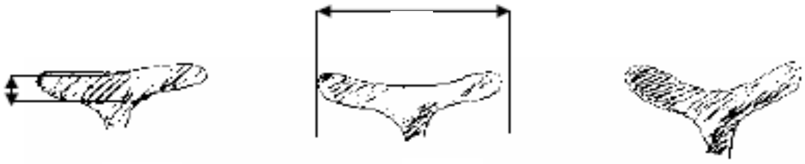

N° GIBA	N° CPVO/ UPOV	CARATTERE	LIVELLO D'ESPRESSIONE DEL CARATTERE		VARIETA' DI RIFERIMENTO
51	51	TENDENZA A PRODURRE GETTI LATERALI DALLA BASE	3	debole	Blanc Hyerois, Castel, Vertu
			~~5~~	media	Violet de Provence, Chrysanthème, Popvert
			7	forte	Cacique, Calico

Note

FOGLIE: se non diversamente indicato, tutte le osservazioni vanno eseguite su foglie completamente sviluppate, sul terzo o quarto giro di foglie a partire dalla base, quando il capolino è di circa 3 cm di diametro.

BRATTEE ESTERNE: tutte le osservazioni vanno effettuate sul quinto giro delle brattee a partire dalla base del capolino centrale.

BRATTEE INTERNE: tutte le osservazioni vanno essere eseguite sul capolino centrale.
